# End-to-end system for rapid and sensitive early-detection of SARS-CoV-2 for resource-poor and field-test environments using a $51 lab-in-a-backpack

**DOI:** 10.1371/journal.pone.0259886

**Published:** 2022-01-26

**Authors:** E. Emily Lin, Umar A. Razzaque, Stephen A. Burrows, Stoyan K. Smoukov

**Affiliations:** School of Engineering and Materials Science, Queen Mary University of London, London, United Kingdom; Waseda University: Waseda Daigaku, JAPAN

## Abstract

COVID-19 has exposed stark inequalities between resource-rich and resource-poor countries. International UN- and WHO-led efforts, such as COVAX, have provided SARS-CoV-2 vaccines but half of African countries have less than 2% vaccinated in their population, and only 15 have reached 10% by October 2021, further disadvantaging local economic recovery. Key for this implementation and preventing further mutation and spread is the frequency of voluntary [asymptomatic] testing. It is limited by expensive PCR and LAMP tests, uncomfortable probes deep in the throat or nose, and the availability of hardware to administer in remote locations. There is an urgent need for an inexpensive “end-to-end” system to deliver sensitive and reliable, non-invasive tests in resource-poor and field-test conditions. We introduce a non-invasive saliva-based LAMP colorimetric test kit and a $51 lab-in-a-backpack system that detects as few as 4 viral RNA copies per μL. It consists of eight chemicals, a thermometer, a thermos bottle, two micropipettes and a 1000–4000 rcf electronically operated centrifuge made from recycled computer hard drives (CentriDrive). The centrifuge includes a 3D-printed rotor and a 12 V rechargeable Li-ion battery, and its 12 V standard also allows wiring directly to automobile batteries, to enable field-use of this and other tests in low infrastructure settings. The test takes 90 minutes to process 6 samples and has reagent costs of $3.5 per sample. The non-invasive nature of saliva testing would allow higher penetration of testing and wider adoption of the test across cultures and settings (including refugee camps and disaster zones). The attached graphical procedure would make the test suitable for self-testing at home, performing it in the field, or in mobile testing centers by minimally trained staff.

## Introduction/Background

The coronavirus disease 2019 (COVID-19) pandemic now has over 240 million confirmed cases worldwide, increasing at over half a million new cases per day during the second wave of the pandemic, and resulting in over 5 million deaths by the end of Oct 2021 [1]. International efforts, such as UN- and WHO-led COVAX are encouraging, but full vaccination is still 2–3 years away. There are real concerns about overwhelming the health-care systems in many countries. With over half of 54 countries reaching less than 2% only 15 countries reaching 10% COVID-19 vaccination by October 2021, the goal of protective vaccination in Africa will be one for the future [2]. Cheap and fast testing is needed in both home environments and point-of-care (POC) facilities, and in both resource-rich and resource-poor countries, for accurately distinguishing COVID-19 from other respiratory illnesses [3, 4]. Such a testing system is needed for safeguarding front-line healthcare workers, for identifying and contact-tracing of infections, for enabling travel, and for providing more measured pandemic responses where limited test administration necessitates complete lockdowns [5]. Similar per-test costs as in the UK are supplemented by extraordinary costs for on-site personnel at US$ 5000–6000 / person per day in California [6].

In addition to the high costs, the invasiveness and pain of the test itself puts up extra barriers to compliance and frequent testing. Currently, the gold standard for COVID-19 detection [7–9], the PCR test requires the uncomfortable feeling of jabbing probes deep into the nostrils, turning people away from testing. To improve experience, speed, and cost, other methods are starting to be used around the world [10, 11]. The reverse transcription loop-mediated isothermal amplification (RT-LAMP) is a one-step nucleic acid amplification method that has a similar sensitivity to the multi-cycle PCR [12, 13] and can be performed on saliva samples [14, 15]. Since the pioneering work of Notomi [14] it is widely used in the diagnosis of infectious diseases [16–21]. The LAMP reaction of DNA amplification at constant temperature has been combined in an assay with a reverse transcription process (RT-LAMP) to target and detect RNA sequences, specifically also the SARS-CoV-2 RNA [22–25]. It is a high sensitivity and high specificity test, and its minimal requirements for equipment and reagents make it a suitable candidate for economic and rapid viral detection on site. Still, such 120-min on-site “fly-safe” LAMP tests at Heathrow cost £85 ($118) and more [26]. Additionally, such tests assume the availability of high value instruments, such as centrifuges, or fluorescence readers, making them inappropriate for in-home testing and remote locations. For such environments, the low-cost of the entire system must be considered too.

Inexpensive tests carried out in residential facilities can increase testing compliance. Furthermore so would non-invasive tests in countries and cultures where the cost or fear of the procedure would prevent testing [27, 28]. In addition to the marginal cost per sample, for many lower-volume sites, one has to also consider the additional cost of infrastructure and staff training. Low-cost approaches have been advocated for both RT-LAMP and RT-qPCR tests [29].

These range from extremely low-cost ($10) device sets that could be operated even without electricity [30], to more expensive $300 equipment that can be used to run concurrent tests in 96-well plates and then reading out fluorescent results with a UV source and a smart phone or a tablet [29]. The appropriate (fixed capital) cost for such instruments depends strongly on the required number of tests to be run at a time, longevity and stable operation of the equipment, and situation-specific factors. Such factors include the distance of test subjects from other amenities, e.g. ability to charge via grid or automobile batteries, refrigeration for RNA primers, etc. Education and training of workers is another component of the fixed (human) capital costs of these tests. Therefore, procedures should be easy to follow, even with only basic literacy skills and minimal training, and ideally with instructions in multiple languages. Thus to maximize the availability and impact of COVID-19 testing around the world, instruments and protocols need to be tailored to situations to minimize local testing system cost [31].

**Fig 1:**
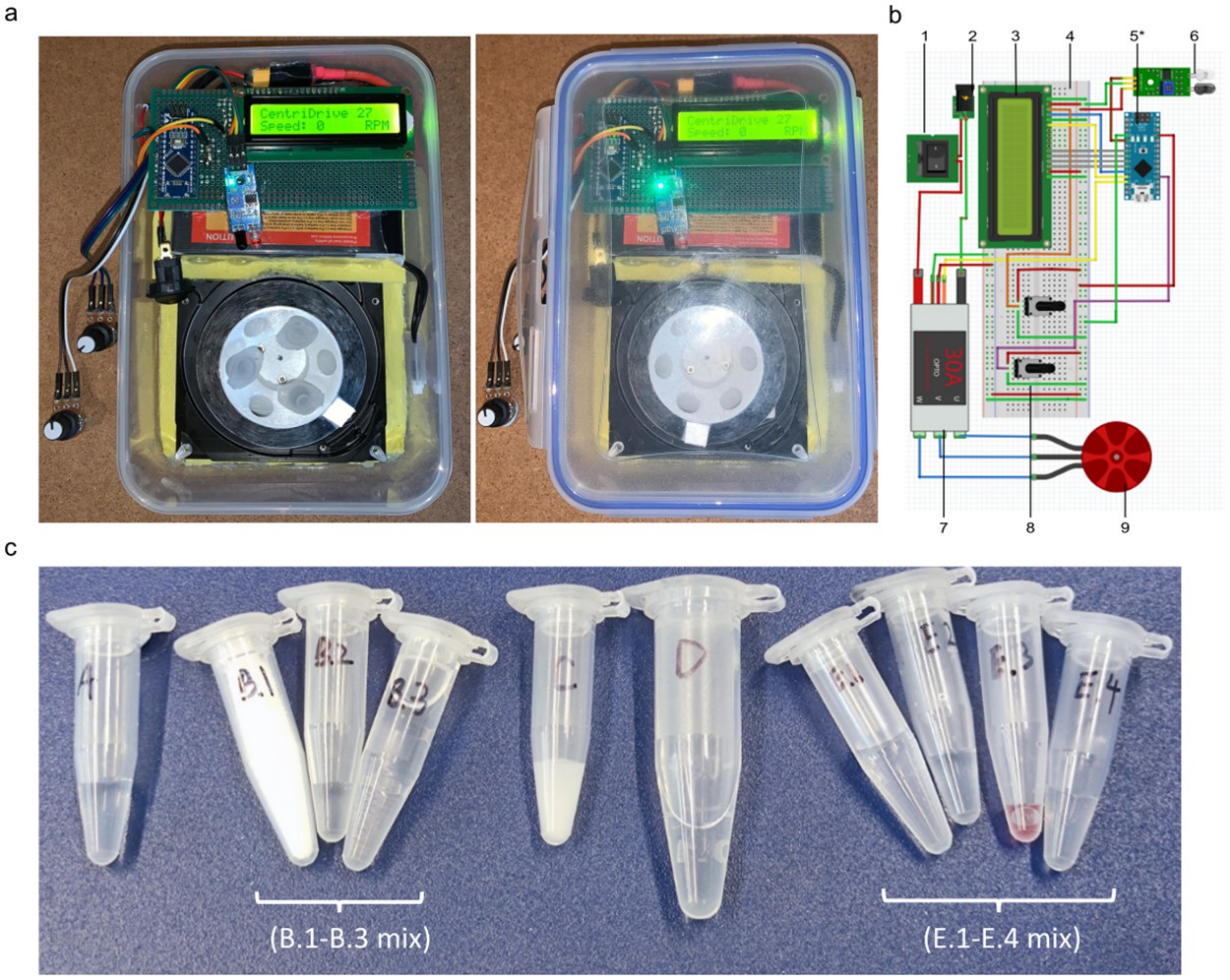
CentriDrive and reagents for LAMP assay. (a) The CentriDrive. (b) Circuit diagram of the CentriDrive. 1. Switch. 2. Power input. 3. LCD. 4. Breadboard. 5. Geekcreit Nano. 6. Infrared sensor. 7. ESC. 8. Potentiometer. 9. Motor. (c) List of all reagents for LAMP assay. Chemical A, Lysis/Inactivation buffer. Chemical B.1, NaI powder. Chemical B.2, 1 N HCl. Chemical B.3, Triton X-100. Chemical C, Silica binding suspension. Chemical D, 80% ethanol solution. Chemical E.1, PBS buffer. Chemical E.2, Primer solution. Chemical E.3, WarmStart^®^ Colorimetric LAMP 2X Master Mix. Chemical E.4, SARS-CoV-2 solution. *The Arduino nano is shown in the figure due to limitations of the drawing software, but the Geekcreit Nano was used in the final test.

Here we report a non-invasive, rapid, low-cost system for COVID-19 testing to replace or complement PCR tests. It consists of a $51 lab-in-a-backpack system (including a high-performance $28 electronic centrifuge made from recycled waste computer hard drives, a thermometer, a thermos bottle, and $20 for two micropipettes), and an RT-LAMP protocol from Harvard Medical School [32] integrated into a single test kit that can be used reliably and reproducibly at home or in remote field conditions (Fig 1). The system instructions include a visual protocol with disposable reagent packs, enabled by graphical instructions for mixing numbered, color-coded packages which can be followed by minimally trained staff. It takes around 80 minutes to test one sample, or 90 minutes to test 6 samples. The test achieves SARS-CoV-2 detection that costs in reagents and consumables approximately $3.5 per sample. Such a widely available non-invasive, rapid, sensitive, and reliable test for COVID has the potential to alleviate stresses in many health care systems.

## Equipment/Materials and methods

### Components for the CentriDrive

The CentriDrive (Fig 1a) is an inexpensive medical device, assembled from low-cost new and recycled components at an approximate cost of $28. In addition to the current use in the covid test, this centrifuge has been used for the demonstration of DNA separation in UK high schools. This low-cost device can be used in a wide variety of resource-poor settings. It can be assembled in local workshops and enterprises using the wiring diagram in Fig 1b along with the following tools: a soldering iron, a T6 screwdriver. The major components are listed below.

### Spinning hard drive

Hard drives with rotating spindles are engineered so well that usually they are disposed with unwanted computers in working order. In cloud computing farms, where they are used constantly, their failure rate is only 1–2% per year on average. Of the non-functioning hard drives the vast majority fail due to head damage or plate seizure. Therefore, after dismantling the head arm and plate (for separate disposal, removing any data/privacy concerns), the motor, controls, and chassis of the hard drive can be used for a centrifuge. The spindle motors in hard drives are designed to operate at high speeds, having a long lifespan of 3–5 years on average. They are well balanced and control their speed of rotation well, making them ideal for the diagnostic centrifuge.

### 3D printed centrifuge rotor

The rotor was designed to hold 6 x 1.5 mL microfuge tubes and has a rotor radius of 3.5 cm. It is printed from 22 g of PLA (polylactic acid) plastic filament using an Ultimaker 3.0, with printing taking approximately 4 hours. The rotor is mounted to the motor with the screws that initially held the plate. Using a commonly available standard 7200 rpm hard drive, the hard drive centrifuge can reach a maximum speed of 11650 rpm (5320 rcf) and 7600 rpm (2260 rcf) when loaded with the microfuge tubes. With 10000 rpm server hard drives, it can reach over 4000 rcf. The speed is controlled by a potentiometer, measured by an IR sensor mounted within range of the rotor, and displayed on an LCD (liquid crystal display) driven by a microcontroller Geekcreit Nano. The consistency of the CentriDrive speed was tested every minute for 15 minutes (See Fig S7 in S1 File).

### Other electronic components (commercially available)

Generic descriptions of components are given, with more details, costs, and sources listed in the Supplementary Material. Components: 5–12 V brushless DC (BLDC), 30 A electronic speed controller (ESC), Geekcreit Nano, 10K potentiometers, 12 V power supply, a rocker switch, and an infrared sensor.

### Materials for the detection

TCEP-HCl, TritonX-100 were purchased from Tokyo Chemical Industry. EDTA, NaI, silicon dioxide, K_2_HPO_4_, KH_2_PO_4_, and nuclease-free water were purchased from Sigma-Aldrich Company. Primers were purchased from Thermo-Fisher. SARS-CoV-2 Template viral RNA was purchased from Twist Bioscience. WarmStart^®^ Colorimetric LAMP 2X Master Mix was purchased from New England Biolabs. All chemicals were of reagent grade and were used without further purification.

### Modified RT-LAMP reaction components [32]

#### Chemical A. Lysis/Inactivation buffer

To a 1.5 mL centrifuge tube, 0.5 mL 0.5 M TCEP-HCl, 0.1 mL 0.5 M EDTA (pH = 8), 0.115 mL 10 N NaOH, and 0.285 mL tap water were added to form 1 mL of the 100X inactivation buffer. Stored at 4°C for later use.

#### Chemical B. NaI binding solution

To a 10 mL tube, Chemical B.1 450 mg NaI power and 4 mL water were added to form 10 wt% NaI solution, then Chemical B.2 50 μL 1 N HCl, Chemical B.3 100 μL TritonX-100 were added to form the Chemica B NaI binding solution. The Chemical B should be freshly prepared before testing.

#### Chemical C. Silica binding suspension

To a 100 mL centrifuge tube, added 5 g 325 mesh silicon dioxide and 50 mL water. Pour off the supernatant after the silica has settled for 1 hour. Another portion of 50 mL water was added to make the silica suspension. This suspension works in conjunction with the NaI binding solution, added in ratios of 50:1.

#### Chemical D. 80% ethanol solution

The ethanol washing solution was made by diluting the 80 mL of absolute ethanol with 20 mL of water.

#### Chemical E.1 PBS buffer

Phosphate-buffered saline (PBS) buffer was made by dissolving 0.2798 g K_2_HPO_4_ and 0.0205 g KH_2_PO_4_ to 50 mL water to make the pH ≈7.8. If the pH is too high, it won’t show the color change from pink to yellow. If the pH is too low, the color change would not be easy to distinguish. Store at 4°C for later use.

#### Chemical E.2 primer solution

The primers (Table 1) were dissolved with nuclease-free water or tap water separately. The sequences are from Rabe and Cepko 2020 [32]. The primer mix were combined with 16 μL FIP, 16 μL BIP, 2 μL F3, 2 μL B3, 4 μL LF, 4 μL LB, and brought to 100 μL with water to make the 10X solution. They were stored at 4°C for later use.

**Table 1:** Sequence of custom primers from 5’ to 3’.

Primer	Sequence (5′– 3′)
F3	CGGTGGACAAATTGTCAC
B3	CTTCTCTGGATTTAACACACTT
FIP	TCAGCACACAAAGCCAAAAATTTATCTGTGCAAAGGAAATTAAGGAG
BIP	TATTGGTGGAGCTAAACTTAAAGCCCTGTACAATCCCTTTGAGTG
LF	TTACAAGCTTAAAGAATGTCTGAACACT
LB	TTGAATTTAGGTGAAACATTTGTCACG

#### Chemical E.3 WarmStart^®^ colorimetric LAMP 2X master mix

Used as purchased and stored at -20°C. The mix is a special low-buffer reaction solution containing phenol red as a visible pH indicator for rapid and easy detection of reverse transcription loop-mediated isothermal amplification (RT-LAMP) reactions [33]. The whole system is designed to provide a fast, clear visual detection of amplification based on the production of acid and subsequent drop in pH [34], producing a change in solution color from pink to yellow [35].

#### Chemical E.4 template viral RNA

Diluted with both nuclease-free water and tap water, separately. Stored at -80°C for later use. Although we used this template viral RNA as positive reference, it doesn’t need to be used in the real test.

### Ethics statement

For solutions where saliva was used in mixture with buffers, only authors’ own self-collected saliva was used. After experiment it was safely discarded as biowaste. The protocol was deemed not to require approval by the Queen Mary University Ethics of Research Committee.

## Results

**Fig 2:**
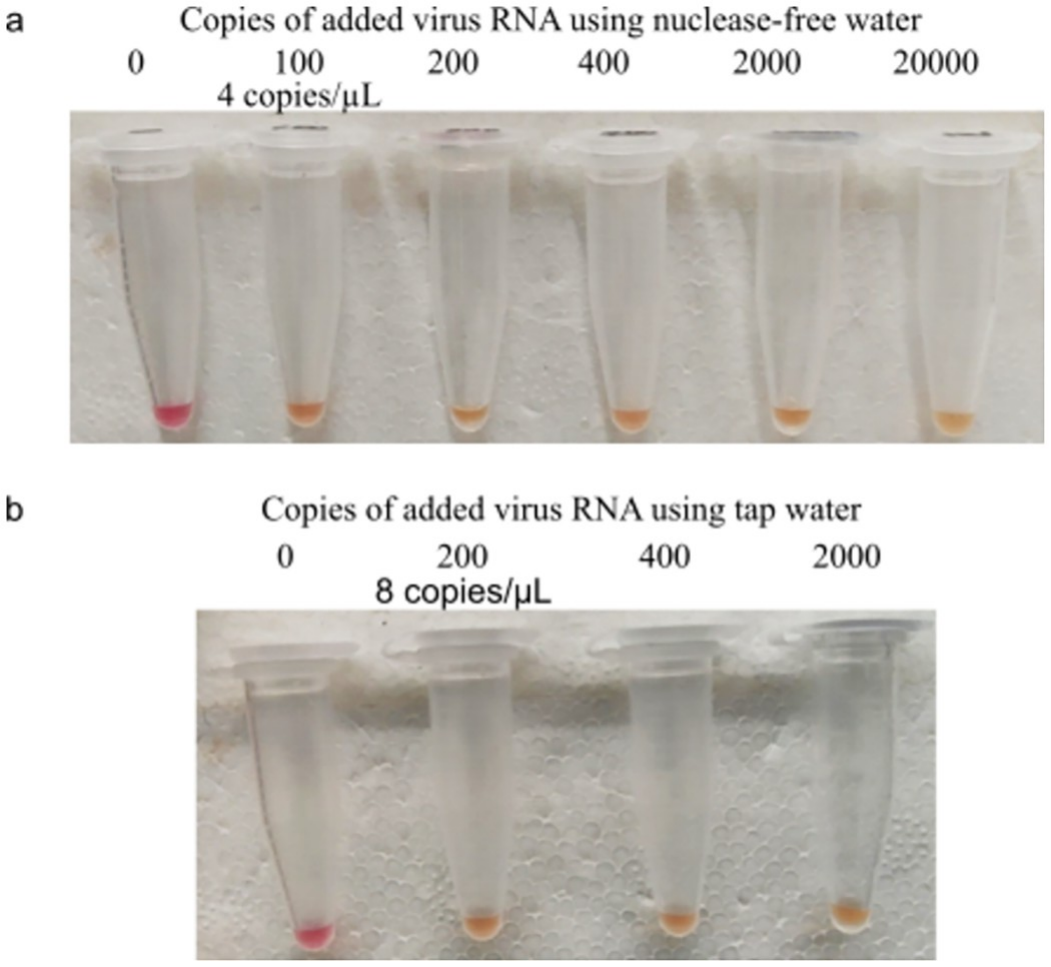
LAMP reaction results. After centrifugation, the LAMP protocol can effectively detect synthetic viral RNA down to 4 copies per μL in saliva. (a) The tubes show the successful amplification of 100, 200, 400, 2000, 20000 copies of the COVID control RNA genomes, respectively. The leftmost sample shows the control color in the absence of LAMP reaction with 0 COVID control RNA genomes. (b) Control and viral RNA samples amplified by LAMP reaction using tap water give comparable results to nuclease-free water.

SARS-CoV-2 RNA was mixed with saliva at varying concentrations and tested with the CentriDrive system to determine the sensitivity of virus detection. The results showed that the RT-LAMP reaction, after the centrifugation, could detect SARS-CoV-2 at 4 viral genome copies/μL. The experiment was repeated nine times, and in all cases the detection was successful. The control experiment contained no virus SARS-CoV-2 RNA and produced no color change of the LAMP solution (Fig 2). Though we usually used nuclease-free water to make the stock solution of the SARS-CoV-2 RNA, we verified that for detection of virus RNA soon after collection, tap water achieved a sensitivity of 8 copies/μL. On both the CentriDrive and the commercial centrifuge (SciSpin—Micro, Cat. No:911315410035) tests, the lowest concentration detected was 4 viral genome copies/μL. The illustrated procedure uses minimal hardware and an easy-to-follow procedure that could be followed both at home and in field point-of-care (POC) centers.

### Detection procedure

Collect around 500 μL of saliva in a 1.5 mL centrifuge tube. It should be ensured that participants do not chew gum, smoke, eat or drink for 30 minutes before submitting the sample. Add 5 μL Chemical A (Lysis inactive solution), mix them and put the tube into boiling water and incubate for 5 minutes, then use the CentriDrive to spin for 1 minute at the maximum speed (~7600 rpm). Collect the supernatant into a new 1.5 mL centrifuge tube.

To the supernatant solution, add 250 μL of Chemical B (NaI binding reagent) and 5 μL of Chemical C (silica slurry), mix them and incubate at room temperature for 10 minutes. Then centrifuge for 30 seconds and pour off the supernatant and keep the precipitation.

Add 700 μL of Chemical D (80% ethanol) to the tube with the precipitation, mix them and centrifuge for 30 seconds at the maximum speed. Pour off the ethanol and keep the lid open to evaporate the residual ethanol at room temperature. Do not try to heat higher than 70°C to speed up the evaporation process, as it might cause RNA degradation. Do not use gas to blow on the tube which may blow the RNA away.

**Fig 3:**
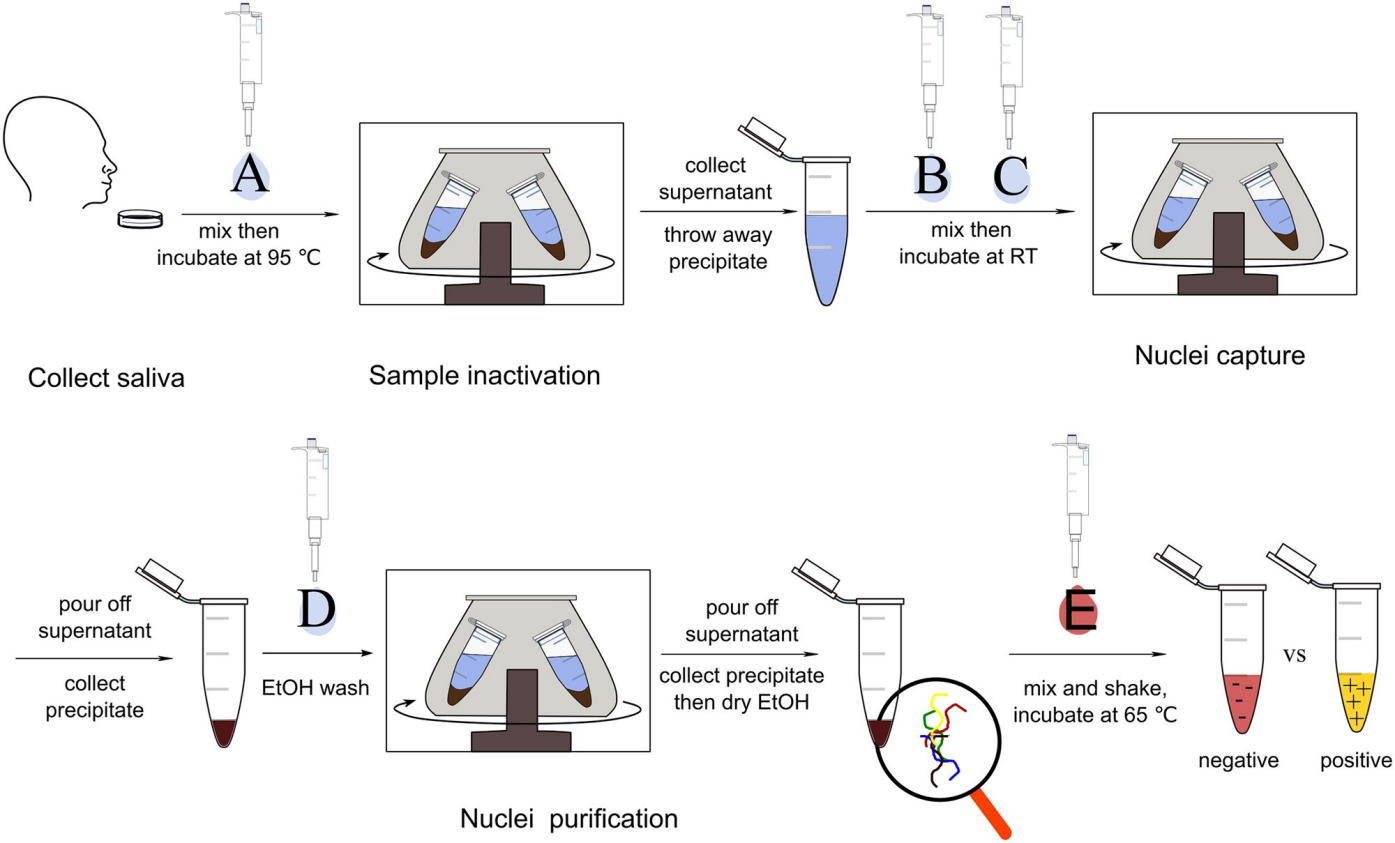
Methods and steps for CentriDrive-LAMP reaction essay process. The following reagents are denoted by letters: A. 5 μL Chemical A; B. 250 μL Chemical B; C. 5 μL Chemical C; D. 700 μL Chemical D; E. 2.5 μL Chemical E.2, 12.5 μL Chemical E.4, Chemical E.1 and Chemical E.3 are 10 μL in total to bring the solution to 25 μL.

To the tube with silica precipitate, add 2.5 μL Chemical E.2 (LAMP primers), 12.5 μL Chemical E.4 (WarmStart^®^ Colorimetric LAMP 2X Master Mix), Chemical E.3 (SARS-CoV-2 solution, add as a positive comparison), and enough Chemical E.1 (PBS solution) to bring the solution to 25 μL, mix them using a vortexer at around 800 rpm/min for 5 seconds or violently shake manually for 30–60 s, followed by centrifuging at 2000 rcf for 30 seconds to ensure all material collects at the bottom of the tube. Otherwise, the reaction may not happen. Then incubate the reaction mix at 65°C for 30 minutes. Positive reactions will turn yellow while negative controls should remain pink (Fig 3).

### Mechanism of the LAMP assay

**Fig 4:**
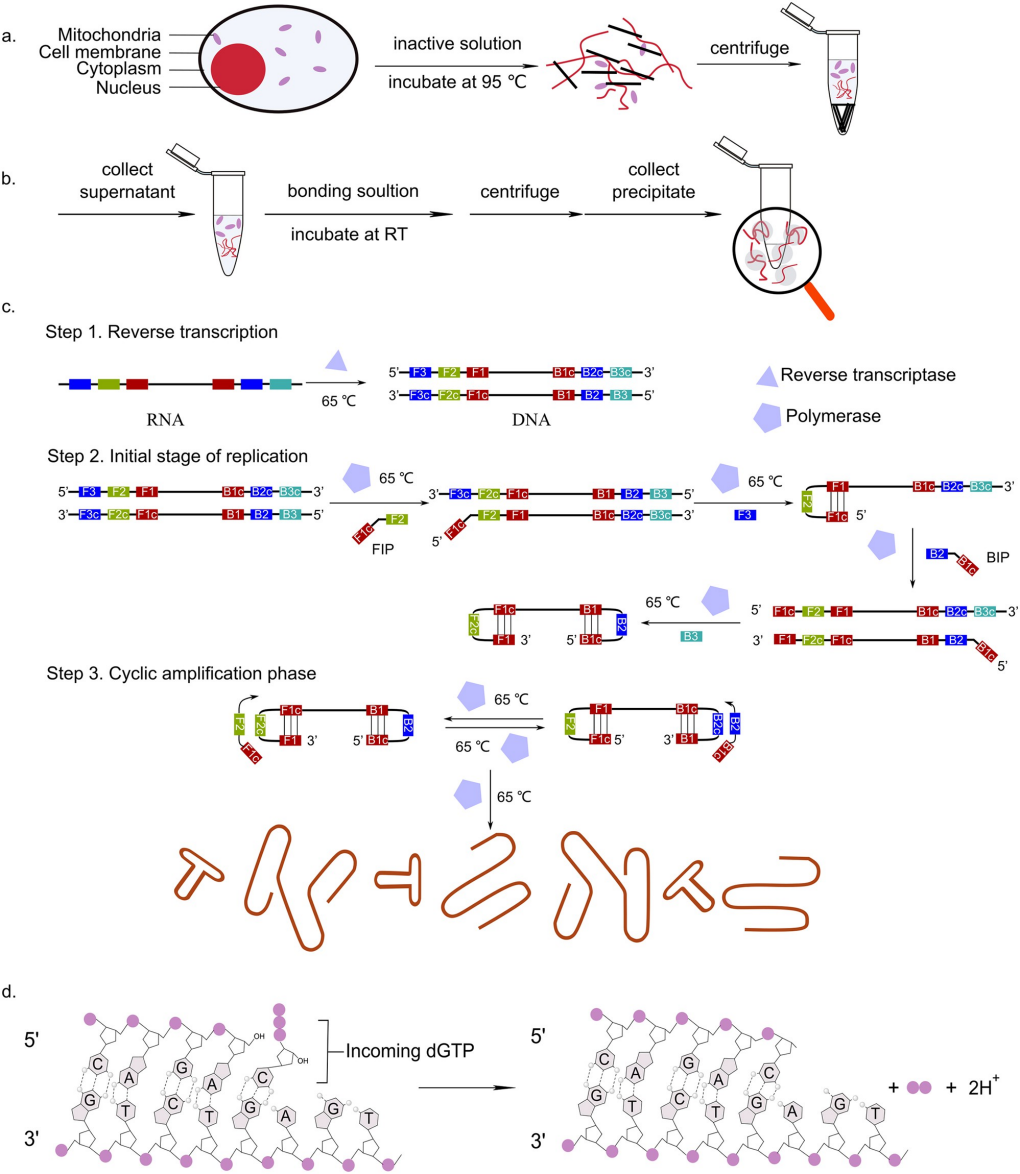
Mechanism of the LAMP assay. (a) Inactivation of the saliva. (b) Binding the nucleic acid. (c) LAMP reaction process. (d) Mechanism of the generation of the proton.

Under alkaline conditions and high temperature, membranes and proteins will be denatured rapidly, while covalently bonded circular plasmid DNA will remain intact in the supernatant. Obtain the required nucleic acid by collecting the supernatant after centrifugation (Fig 4a).

The nucleic acids will bind to the silica in the presence of chaotropic salts, such as NaI used here [36, 37]. Obtain the nucleic acid bound with silica by collecting the precipitate after centrifugation (Fig 4b).

In the LAMP reaction, the target sequence is amplified at a constant temperature of 60–65°C, using three sets of primers) and a polymerase. The first step is the reverse transcription of RNA to DNA in the presence of reverse transcriptase. Then each of the primer possesses a sequence complementary to one chain of the amplification region at the 3’-terminal and identical to the inner region of the same chain at the 5’-terminal, finally forms the dumbbell-like structure [15]. The addition of loop primers to the reaction accelerates the DNA amplification [38]. The amplified products again pass through repeated elongation reactions, which generate amplified DNA products of various stem lengths [39]. (Fig 4c).

The DNA synthesis process is the formation of a phosphodiester bond between the incoming de-oxynucleoside triphosphate and the terminal primer nucleotide catalysed by DNA polymerases. It will release a pyrophosphate (PPi) [40]. (Fig 4d) Hydrolysis of pyrophosphate will generate two hydrogen protons which lower the solution pH and is detected by color change in the pH color indicator.

## Conclusions

By using the LAMP test method in combination with our $51 lab-in-a-backpack system, pictured in Fig S11 (included in S1 File), we can successfully detect SARS-CoV-2 at 4 viral genomes/μL. This detection sensitivity is much lower than found in infected individuals [41] and compares favorably to commercial LAMP tests, which detect 4 viral genomes/μL [42]. Commercial LAMP test kits sold to testing companies cost £5–17 ($7–24) in bulk. The test is fast and cheap taking < 90 minutes at $3.5 per sample. The test and hardware reported here could be the basis of a stand-alone system and an end-to-end solution for COVID-19 testing. The centrifuge described above could also be used as part of another commercial LAMP test to complete a hardware system and an end-to-end solution.

## Supporting information

S1 File Supplementary materials. Including Assay time for one sample and six samples, The hard drive centrifuge recipe, The consistence of the CentriDrive speed, Rotational speed characterization of CentriDrive at different voltage, LAMP reaction results using commercial centrifuge instead of the CentriDrive, A prototype kit with vials in several pouches.
(PDF)

S1 Graphical abstract (TIF)

## References

[1] WHO Coronavirus Disease (COVID-19) Dashboard: Word Health Organization; [cited 2021 August 27 and 2021 October 26]. https://covid19.who.int.

[2] Fifteen African countries hit 10% COVID-19 vaccination goal: Word Health Organization; [cited 2021 Oct 26]. https://www.afro.who.int/news/fifteen-african-countries-hit-10-covid-19-vaccination-goal.

[3] Songok E. A locally sustainable approach to COVID-19 testing in Africa. The Lancet Microbe. 2020;1(5). doi: 10.1016/S2666-5247(20)30118-X 33521721 PMC7836952

[4] Adepoju P. Africa’s struggle with inadequate COVID-19 testing: The Lancet Microbe; 2020 [cited 2021 March 21]. https://www.thelancet.com/journals/lanmic/article/PIIS2666-5247(20)30014-8/fulltext. 10.1016/S2666-5247(20)30014-8 PMC7212979 32835324

[5] Wang YC, Lee YT, Yang T, Sun JR, Shen CF, Cheng CM. Current diagnostic tools for coronaviruses–From laboratory diagnosis to POC diagnosis for COVID-19. Bioengineering & Translational Medicine. 2020. doi: 10.1002/btm2.10177 32838038 PMC7435577

[6] STANDARD AGREEMENT. 2020 [cited 2021 March 21]. https://files.covid19.ca.gov/pdf/Verily-life-sciences-LLC-OES-6155.pdf.

[7] Tahamtan A, Ardebili A. Real-time RT-PCR in COVID-19 detection: issues affecting the results. Expert Review of Molecular Diagnostics. 2020;20(5):453–4. doi: 10.1080/14737159.2020.1757437 32297805 PMC7189409

[8] Oliveira BA, Oliveira LCD, Sabino EC, Okay TS. SARS-CoV-2 and the COVID-19 disease: a mini review on diagnostic methods. Revista do Instituto de Medicina Tropical de São Paulo. 2020;62. doi: 10.1590/S1678-9946202062044 32609256 PMC7325591

[9] Bustin SA, Nolan T. RT-qPCR Testing of SARS-CoV-2: A Primer. International Journal of Molecular Sciences. 2020;21(8):3004. doi: 10.3390/ijms21083004 32344568 PMC7215906

[10] Ward S, Lindsley A, Courter J, Assa’ad A. Clinical testing for COVID-19. J Allergy Clin Immunol. 2020;146(1):23–34. Epub 2020/05/24. doi: 10.1016/j.jaci.2020.05.012 . 32445839 PMC7237919

[11] Chau CH, Strope JD, Figg WD. COVID-19 Clinical Diagnostics and Testing Technology. Pharmacotherapy: The Journal of Human Pharmacology and Drug Therapy. 2020;40(8):857–68. doi: 10.1002/phar.2439 32643218 PMC7361586

[12] Nagura-Ikeda M, Imai K, Tabata S, Miyoshi K, Murahara N, Mizuno T, et al. Clinical Evaluation of Self-Collected Saliva by Quantitative Reverse Transcription-PCR (RT-qPCR), Direct RT-qPCR, Reverse Transcription–Loop-Mediated Isothermal Amplification, and a Rapid Antigen Test To Diagnose COVID-19. Journal of Clinical Microbiology. 2020;58(9). doi: 10.1128/jcm.01438-20 32636214 PMC7448663

[13] Baek YH, Um J, Antigua KJC, Park JH, Kim Y, Oh S, et al. Development of a reverse transcription-loop-mediated isothermal amplification as a rapid early-detection method for novel SARS-CoV-2. Emerg Microbes Infect. 2020;9(1):998–1007. Epub 2020/04/21. doi: 10.1080/22221751.2020.1756698 . 32306853 PMC7301696

[14] Notomi T, Okayama H, Masubuchi H, Yonekawa T, Watanabe K, Amino N, et al. Loop-mediated isothermal amplification of DNA. Nucleic acids research. 2000;28(12):e63–e. doi: 10.1093/nar/28.12.e63 10871386 PMC102748

[15] Notomi T, Mori Y, Tomita N, Kanda H. Loop-mediated isothermal amplification (LAMP): principle, features, and future prospects. J Microbiol. 2015;53(1):1–5. Epub 2015/01/06. doi: 10.1007/s12275-015-4656-9 . 25557475

[16] Fukuta S, Iida T, Mizukami Y, Ishida A, Ueda J, Kanbe M, et al. Detection of Japanese yam mosaic virus by RT-LAMP. Arch Virol. 2003;148(9):1713–20. Epub 2003/09/25. doi: 10.1007/s00705-003-0134-5 . 14505084

[17] Aonuma H, Yoshimura A, Kobayashi T, Okado K, Badolo A, Nelson B, et al. A single fluorescence-based LAMP reaction for identifying multiple parasites in mosquitoes. Exp Parasitol. 2010;125(2):179–83. Epub 2010/01/13. doi: 10.1016/j.exppara.2009.12.023 . 20064511

[18] Fukuta S, Takahashi R, Kuroyanagi S, Miyake N, Nagai H, Suzuki H, et al. Detection of Pythium aphanidermatum in tomato using loop-mediated isothermal amplification (LAMP) with species-specific primers. European Journal of Plant Pathology. 2013;136(4):689–701. doi: 10.1007/s10658-013-0198-3

[19] Caipang CM, Haraguchi I, Ohira T, Hirono I, Aoki T. Rapid detection of a fish iridovirus using loop-mediated isothermal amplification (LAMP). J Virol Methods. 2004;121(2):155–61. Epub 2004/09/24. doi: 10.1016/j.jviromet.2004.06.011 . 15381352

[20] Hsieh K, Mage PL, Csordas AT, Eisenstein M, Soh HT. Simultaneous elimination of carryover contamination and detection of DNA with uracil-DNA-glycosylase-supplemented loop-mediated isothermal amplification (UDG-LAMP). Chem Commun (Camb). 2014;50(28):3747–9. Epub 2014/03/01. doi: 10.1039/c4cc00540f . 24577617

[21] Duan Y, Ge C, Zhang X, Wang J, Zhou M. A rapid detection method for the plant pathogen Sclerotinia sclerotiorum based on loop-mediated isothermal amplification (LAMP). Australasian Plant Pathology. 2013;43(1):61–6. doi: 10.1007/s13313-013-0239-6

[22] Yu L, Wu S, Hao X, Li X, Liu X, Ye S, et al. Rapid colorimetric detection of COVID-19 coronavirus using a reverse tran-scriptional loop-mediated isothermal amplification (RT-LAMP) diagnostic plat-form: iLACO. medRxiv. 2020. 10.1093/clinchem/hvaa102 PMC7188121 32315390

[23] Zhang Y, Odiwuor N, Xiong J, Sun L, Nyaruaba RO, Wei H, et al. Rapid Molecular Detection of SARS-CoV-2 (COVID-19) Virus RNA Using Colorimetric LAMP. medRxiv. 2020:2020.02.26.20028373. doi: 10.1101/2020.02.26.20028373

[24] Kashir J, Yaqinuddin A. Loop mediated isothermal amplification (LAMP) assays as a rapid diagnostic for COVID-19. Med Hypotheses. 2020;141:109786. Epub 2020/05/04. doi: 10.1016/j.mehy.2020.109786 . 32361529 PMC7182526

[25] Thompson D, Lei Y. Mini review: Recent progress in RT-LAMP enabled COVID-19 detection. Sensors and Actuators Reports. 2020;2(1). doi: 10.1016/j.snr.2020.100017 PMC7428436 35047828

[26] Heathrow Airport. 2021. Covid 19 test: Heathrow; 2021 [cited 2021 August 27]. https://www.heathrow.com/at-the-airport/fly-safe/covid-19-test.

[27] Pasomsub E, Watcharananan SP, Boonyawat K, Janchompoo P, Wongtabtim G, Suksuwan W, et al. Saliva sample as a non-invasive specimen for the diagnosis of coronavirus disease 2019: a cross-sectional study. Clinical Microbiology and Infection. 2020. doi: 10.1016/j.cmi.2020.05.001 32422408 PMC7227531

[28] Vaz SN, Santana DSD, Netto EM, Pedroso C, Wang W-K, Santos FDA, et al. Saliva is a reliable, non-invasive specimen for SARS-CoV-2 detection. The Brazilian Journal of Infectious Diseases. 2020;24(5):422–7. doi: 10.1016/j.bjid.2020.08.001 32888905 PMC7458056

[29] Arumugam A, Faron ML, Yu P, Markham C, Wong S. A Rapid COVID-19 RT-PCR Detection Assay for Low Resource Settings. 2020. doi: 10.1101/2020.04.29.069591 PMC7598596 32987722

[30] Li E, Larson A, Kothari A, Prakash M. Handyfuge-LAMP: low-cost and electricity-free centrifugation for isothermal SARS-CoV-2 detection in saliva. medRxiv. 2020:2020.06.30.20143255. doi: 10.1101/2020.06.30.20143255

[31] Manero A, Smith P, Koontz A, Dombrowski M, Sparkman J, Courbin D, et al. Leveraging 3D Printing Capacity in Times of Crisis: Recommendations for COVID-19 Distributed Manufacturing for Medical Equipment Rapid Response. International Journal of Environmental Research and Public Health. 2020;17(13):4634. doi: 10.3390/ijerph17134634 32605098 PMC7370126

[32] Rabe BA, Cepko C. SARS-CoV-2 detection using isothermal amplification and a rapid, inexpensive protocol for sample inactivation and purification. Proceedings of the National Academy of Sciences. 2020;117(39):24450–8. doi: 10.1073/pnas.2011221117 32900935 PMC7533677

[33] WarmStart^®^ Colorimetric LAMP 2X Master Mix: NEW ENGLAND BioLabs; [cited 2021 March 21]. https://international.neb.com/products/m1800-warmstart-colorimetric-lamp-2x-master-mix-dna-rna.

[34] Mori Y, Kitao M, Tomita N, Notomi T. Real-time turbidimetry of LAMP reaction for quantifying template DNA. Journal of biochemical and biophysical methods. 2004;59(2):145–57. doi: 10.1016/j.jbbm.2003.12.005 15163526

[35] Salk JE, Youngner J, Ward EN. Use of color change of phenol red as the indicator in titrating poliomyelitis virus or its antibody in a tissue-culture system. American journal of hygiene. 1954;60(2):214–30. doi: 10.1093/oxfordjournals.aje.a119714 13197372

[36] Vogelstein B, Gillespie D. Preparative and analytical purification of DNA from agarose. Proceedings of the National Academy of Sciences. 1979;76(2):615–9. doi: 10.1073/pnas.76.2.615 284385 PMC382999

[37] Boom R, Sol CJ, Salimans MM, Jansen CL, Wertheim-Van Dillen PM, Van Der Noordaa J. Rapid and simple method for purification of nucleic acids. Journal of Clinical Microbiology. 1990;28(3):495–503. doi: 10.1128/jcm.28.3.495-503.1990 1691208 PMC269651

[38] Nagamine K, Hase T, Notomi T. Accelerated reaction by loop-mediated isothermal amplification using loop primers. Mol Cell Probes. 2002;16(3):223–9. Epub 2002/07/30. doi: 10.1006/mcpr.2002.0415 . 12144774

[39] Hardinge P, Murray JA. Reduced false positives and improved reporting of loop-mediated isothermal amplification using quenched fluorescent primers. Scientific reports. 2019;9(1):1–13. 31089184 10.1038/s41598-019-43817-z PMC6517417

[40] Kottur J, Nair DT. Pyrophosphate hydrolysis is an intrinsic and critical step of the DNA synthesis reaction. Nucleic Acids Research. 2018;46(12):5875–85. doi: 10.1093/nar/gky402 29850882 PMC6159520

[41] Sender R, Bar-On YM, Gleizer S, Bernshtein B, Flamholz A, Phillips R, et al. The total number and mass of SARS-CoV-2 virions. Proceedings of the National Academy of Sciences. 2021;118(25):e2024815118. doi: 10.1073/pnas.2024815118 34083352 PMC8237675

[42] Butler DJ, Mozsary C, Meydan C, Danko D, Foox J, Rosiene J, et al. Shotgun Transcriptome and Isothermal Profiling of SARS-CoV-2 Infection Reveals Unique Host Responses, Viral Diversification, and Drug Interactions. bioRxiv. 2020:2020.04.20.048066. doi: 10.1101/2020.04.20.048066 33712587 PMC7954844

