# Supplementary material for: End-to-end system for rapid and sensitive early-detection of SARS-CoV-2 for resource-poor and field-test environments using a $51 lab-in-a-backpack
Source: PLoS One. 2022 Jan 26;17(1):e0259886. doi: 10.1371/journal.pone.0259886 (PMC8791454; doi:10.1371/journal.pone.0259886)
Supplement: S1 File — Including Assay time for one sample and six samples, The hard drive centrifuge recipe, The consistence of the CentriDrive speed, Rotational speed characterization of CentriDrive at different voltage, LAMP reaction results using commercial centrifuge instead of the CentriDrive, A prototype kit with vials in several pouches. (PDF) [file pone.0259886.s001.pdf]

Contents

1. Assay time for one sample and six samples

2. The hard drive centrifuge recipe

3. The consistence of the CentriDrive speed

4. Rotational speed characterization of CentriDrive at different voltage

5. LAMP reaction results using commercial centrifuge instead of the CentriDrive.

6. A prototype kit with vials in several pouches

Reference

1. Assay time for one sample and six samples

Our lab-in-backpack system can make a maximum of six samples at one time. The difference of assaying one sample and six sample is repeated protocol for adding regents and purifying the nuclei acid. The waiting time for incubation and evaporation and centrifugation are the same.

Table S1. Assay time for one sample and six samples.

Sample number	Assay time/minute
1	80
6	90

2. The hard drive centrifuge Recipe

The CentriDrive (Fig S1) is a diagnostic 3D-printed hard drive centrifuge, at high rotational speeds of up to 10,000 RPM. Most importantly, at a cost of only $28 (See Appendix), this centrifuge is significantly cheaper than a standard centrifuge.

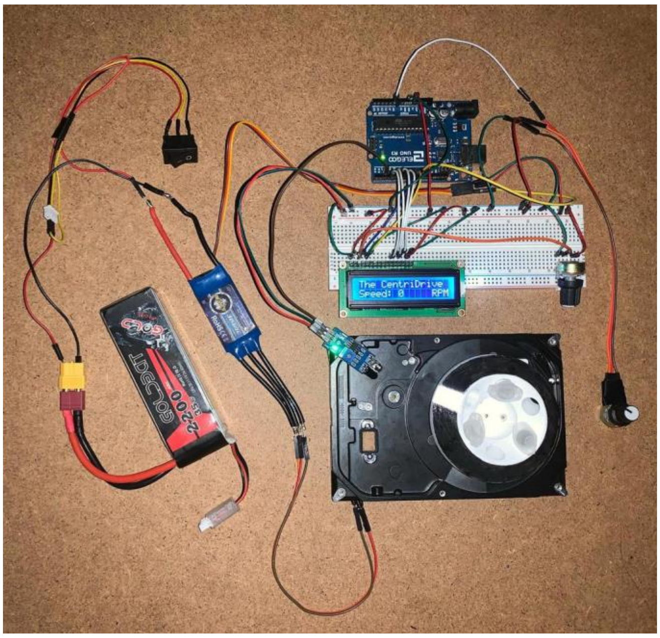

Fig S1: The CentriDrive

Equipment Needed

<u>Parts:</u>

1x 7,200 RPM Hard drive

1x Breadboard

1x Arduino/Geekcreit Nano with USB upload cable

1x 16x2 LCD

2x 10K potentiometers

1x 10-12V battery

1x 30A ESC

1x Rocker switch

1x Centrifuge rotor

1x Infrared sensor

>35x breadboard wires

Tools:

1x T6 Screwdriver

1x Multimeter

1x Soldering Iron

Software:

Arduino IDE – Install from: https://www.arduino.cc/en/main/software

Choose the correct download option for your computer (red arrow Fig S2).

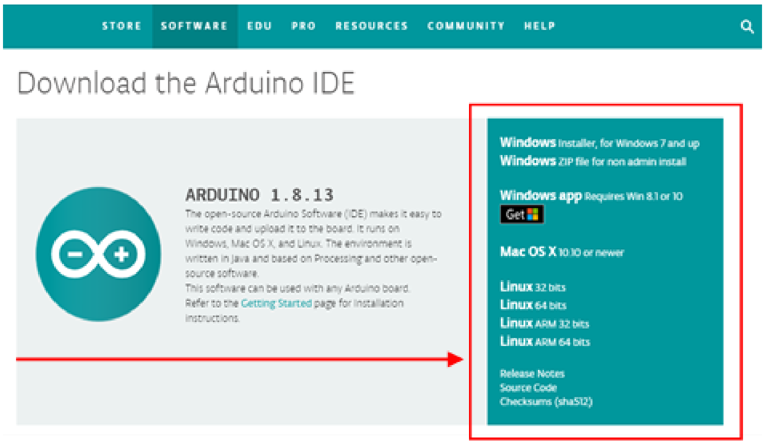

Fig S2. Download Arduino IDE software

Procedure

Step 1: Opening the hard drive

Using the T6 screwdriver, unscrew all T6 screws on the back of the hard drive, including one screw underneath the white label (Fig S3). Once all screws have been removed, use a thin object to remove any glue between the edges. Then remove the back casing to expose the inside of the hard drive.

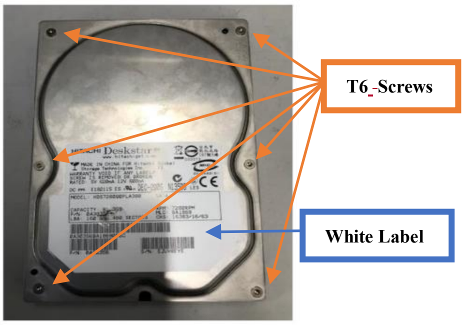

Fig S3: Back face of a hard drive

Step 2: Attaching the rotor

Unscrew the T6 spindle screws located in the centre of the round spindle disk. The platter, spindle disk and actuator arm can now be removed as they are not needed (Fig S4). Screw the 3D-printed centrifuge rotor onto the motor.

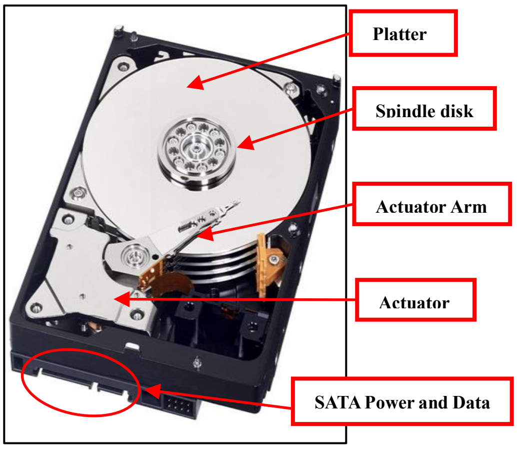

Fig S4: Parts of a hard drive

Step 3: Connecting the motor to the ESC

This step involves the use of the soldering iron and the multimeter. Remove the green processor chip, revealing the motor with either three or four pins. Four-pin motors have three active pins and one common pin. To identify the common pin, measure the resistance between each pin using the multimeter (switch to the 200 Ω setting). The common pin (usually the far-left pin), will have half the resistance across it compared to the three active pins. Solder the three active pins to the three ESC motor pins.

Step 4: Setting up the CentriDrive

By following the CentriDrive schematic shown in Fig S5, connect all the parts together using the appropriate pins (See Appendix) and breadboard wires. Finally, copy and paste the code provided (See Appendix) into the Arduino IDE software and upload it to the Geekcreit Nano via the USB input. Use the physical set-up in Fig S1 for reference.

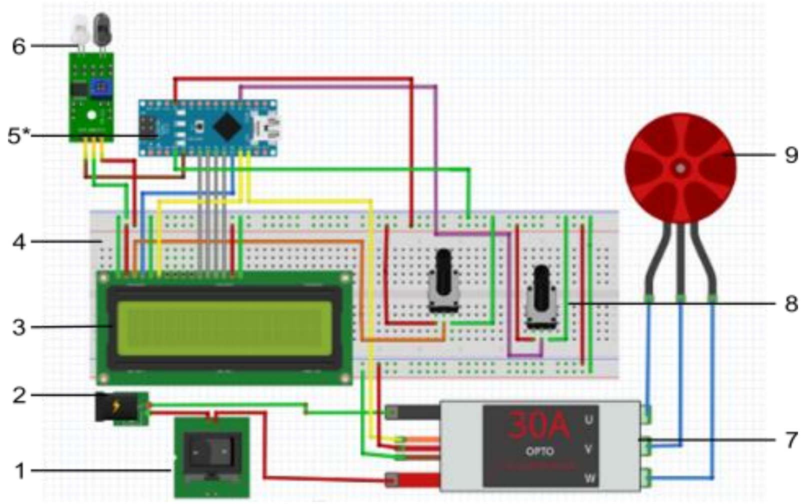

Fig S5. Circuit diagram of the CentriDrive. 1. Switch. 2. Power input. 3. LCD. 4. Breadboard. 5. Geekcreit Nano. 6. Infrared sensor. 7. ESC. 8. Potentiometer. 9. Motor. *The Arduino nano is shown in the figure due to the limitation of the software, but the Geekreit Nano was used in the final test.

Appendix

Table S2: Connecting the LCD

LCD Pin	Name	Connected Pin
1	VSS	GND
2	VCC	5V
3	VEE	Centre pin of potentiometer
4	RS (register select)	Geekcreit Nano pin 8
5	RW (read/write)	GND
6	E (Enable)	Geekcreit Nano pin 9
7	DB0	N/A
8	DB1	N/A
9	DB2	N/A
10	DB3	N/A
11	DB4	Geekcreit Nanopin 4
12	DB5	Geekcreit Nano pin 5
13	DB6	Geekcreit Nano pin 6
14	DB7	Geekcreit Nano pin 7
15	Anode	5V
16	Cathode	GND

Table S3: Connecting the ESC

ESC Pin	Connected Pin
Signal Pin (Yellow)	Geekcreit Nano pin 10
5V	5V
GND	GND

Table S4: Connecting the potentiometer

Potentiometer Pin	Connected Pin
Signal Pin (Middle)	Geekcreit Nano pin A0
5V	5V
GND	GND

Table S5: Connecting the potentiometer

IR sensor	Connected Pin
Signal Pin	Geekcreit Nano pin 2
5V	5V
GND	GND

How to use a breadboard:

https://www.youtube.com/watch?v=6WReFkfrUIk

How an ESC works:

https://www.youtube.com/watch?v=OZNxbxL7cdc&t=597s

Table S6: Costs of each part

CentriDrive part	Cost ($)	Availability
2.85 mm diameter PLA filament	20.58/0.5 kg<br>(22 g mass rotor = 0.91)	Ebay - Rast 3D [eBay item number: 362815622512]
11.1v 2200 mAh Lipo rechargeable battery	9.77	HobbyKing – Rhino [SKU: 9952000024-0]
30 A ESC	4.12	Ebay - G-sun [eBay item number: 353142425598]
Geekcreit Nano	5.04	Amazon – Geekcreit Nano [ASIN: B072BMYZ18]
LCD	4.18	Banggood – Geekcreit [ID: 978160]
2X 10 k Potentiometer	1.38	Amazon – GAOHOU [ASIN: B00H969CM0]
Switch	0.74	CPC Farnell - PRO POWER [catalogue page: CPCC/1490]
35 Wires	1.73	CFC Farnell - PRO SIGNAL [catalogue page: CPCC/1561]

IR Sensor	0.37	AliExpress- https://www.aliexpress.com/item/32321964595.html
**Total Cost**	28.24	

**Pipette part**	**Cost ($)**	**Availability**
2-20 µL	10	Alibaba- https://www.alibaba.com/product-detail/WEST-TUNE-Toppette-Micropipette-0-1ul_1600269219690.html
100-1000 µL	10	Alibaba- https://www.alibaba.com/product-detail/WEST-TUNE-Toppette-Micropipette-0-1ul_1600269219690.html
**Total Cost**	20	

**Thermo part**	**Cost ($)**	**Availability**
Thermos	2.18	Alibaba- https://m.alibaba.com/product/1600271573030/17oz-cola-shape-fitness-thermo-cup.html
Digital thermometer	0.84	Alibaba- https://m.alibaba.com/product/60833309573/Black-Mini-LCD-Display-Electronic-Fish.htmly
**Total Cost**	3.02	

Code:

```
#include <LiquidCrystal.h>  // Includes the LCD library
LiquidCrystal lcd(8, 9, 4, 5, 6, 7);  // LCD pins (RS,E,D4,D5,D6,D7)
#include <Servo.h> // Includes the servo library
Servo esc;  // Stating that esc means Servo
int throttlePin = 0;  // Potentiometer (Pot) connected to Pin A0
void setup() {
esc.attach(10);  // ESC signal pin connected to Pin 10
lcd.begin(16, 2);  // Set up the LCD's number of columns and rows
  lcd.print("Initialising...");  // Print a message to the LCD.
    delay(4000);  // Delay for 2 seconds
      lcd.clear(); // Clear the screen
      lcd.print(" Developed");
      lcd.setCursor(8,1);
      lcd.print("by Umar");
      delay(4000);
```

```
      lcd.clear();
Serial.begin(9600);  // Display on Serial Monitor
}
void loop() {
lcd.clear();
throttlePin = analogRead(A0);  // Read the input on analog pin 0
int throttle = analogRead(throttlePin);  // Read value from Pot
int RPM = (throttlePin*10.23);  // Divide max motor speed by max Pot value
(10532/1023= 10.23)
throttle = map(throttle, 0, 1023, 1000, 2000);  // Map Pot onto ESC
esc.writeMicroseconds(throttle);  // Input Pot values to ESC
lcd.print("The CentriDrive");  // Print a message to the LCD at (0,0)
lcd.setCursor(0, 1);  // Position Text on LCD
 lcd.print("Speed:");
lcd.setCursor(13, 1);
 lcd.print("RPM");
lcd.setCursor(7, 1);
 lcd.print(RPM);  // Display 'RPM' value on LCD Display
Serial.println(RPM);   // Display 'RPM' value on Serial Monitor
  delay(1000);
  lcd.clear();
}
```

Measurement of the Speed via IR Sensor

The CentriDrive uses an infrared (IR) sensor which operates as a digital tachometer to LED, onto a piece of metallic tape and receives the reflected light back using a photodiode (IR Receiver), at the frequency of the motor speed (Fig S6).[1] As part of the CentriDrive system, the IR sensor interacts with the Geekcreit Nano to measure the rotor rotation in revolutions per minute (RPM), displaying the speed on an inbuilt LCD. Similar to the way an encoder operates, the Geekcreit Nano records the time between each received IR ray collected by the IR sensor. The Geekcreit Nano then takes the reciprocal of this measure to obtain revolutions per second (RPS) and then multiplies that value by 60 to get RPM.

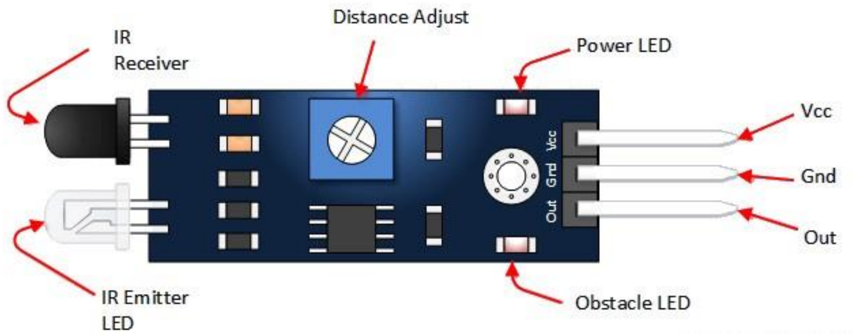

Fig S6. The pin map of the IR Sensor.

Speed data was collected by the IR sensor, processed by the Geekcreit Nano and displayed on the LCD. However, the speed of the rotor could also be displayed directly using the Arduinos integrated development environment (IDE). This data could then be exported from the IDE to a spreadsheet (Excel) and filtered to only show speed values at specific intervals.

A 15-minute centrifuge maximum speed test was conducted at least three times to obtain 3 different data sets. Once the centrifuge was turned on, the potentiometer was turned to full and as soon as the centrifuge hits its maximum speed, it was timed for 15 minutes to investigate its consistency. An average was taken across all three data sets to obtain fifteen individual speed values and plotted on a graph of Speed (RPM) against Time (minutes) at 1-minute intervals. The results showed that the centrifuge did not go below its maximum speed during each 15-minute test, hence proving the consistency of the CentriDrive.

3. The consistency of the CentriDrive speed.

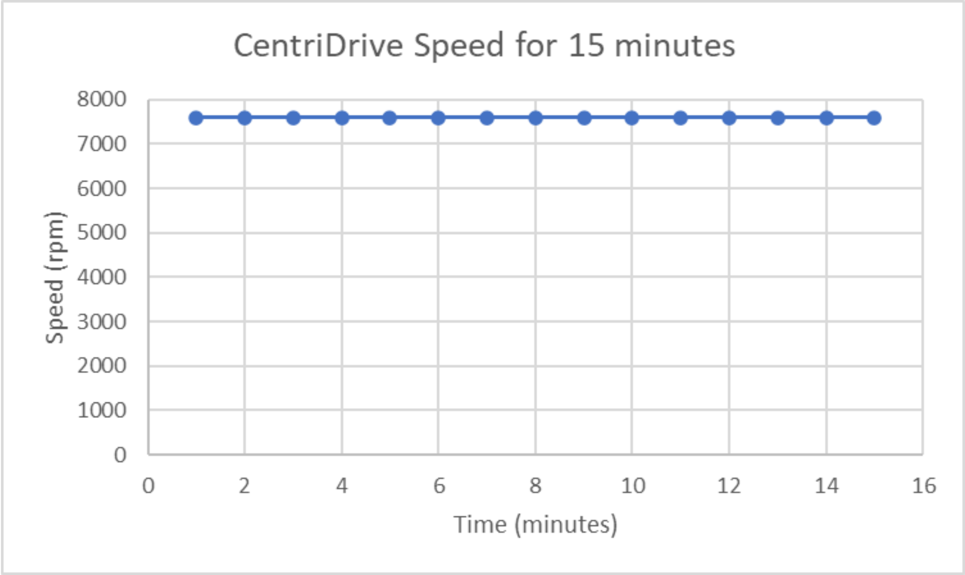

Fig S7. Relationship between the CentriDrive speed and running time. The tests carried out with an attached rotor containing three water-filled microfuge tubes. Once the centrifuge was turned on, the potentiometer was turned to full and as soon as the centrifuge his its maximum speed, it was timed for 15 minutes to investigate its consistency. The error bar is within the size of the symbols.

4. Rotational speed characterization of CentriDrive at different voltages.

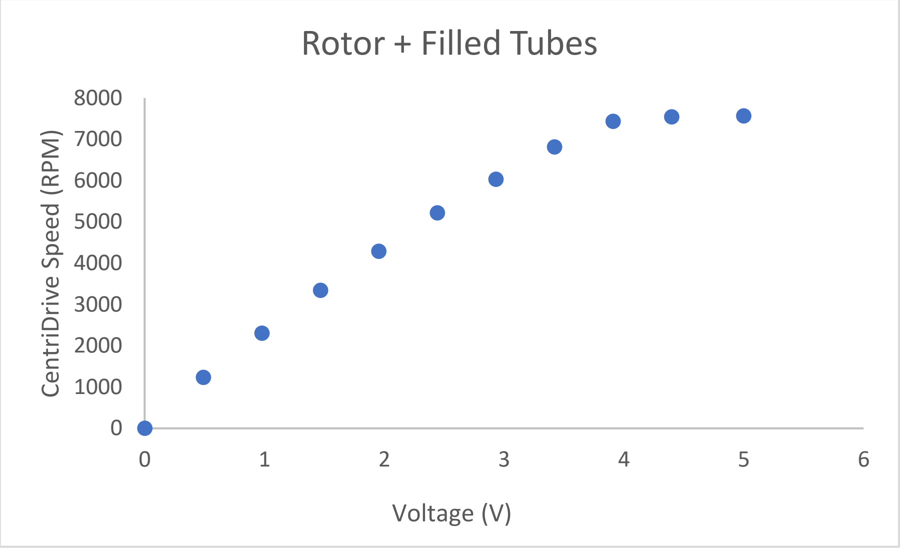

Fig S8. Relationship between the voltage and CentriDrive speed (7200 rpm hard drive base).

5. LAMP reaction results using commercial centrifuge instead of the CentriDrive.

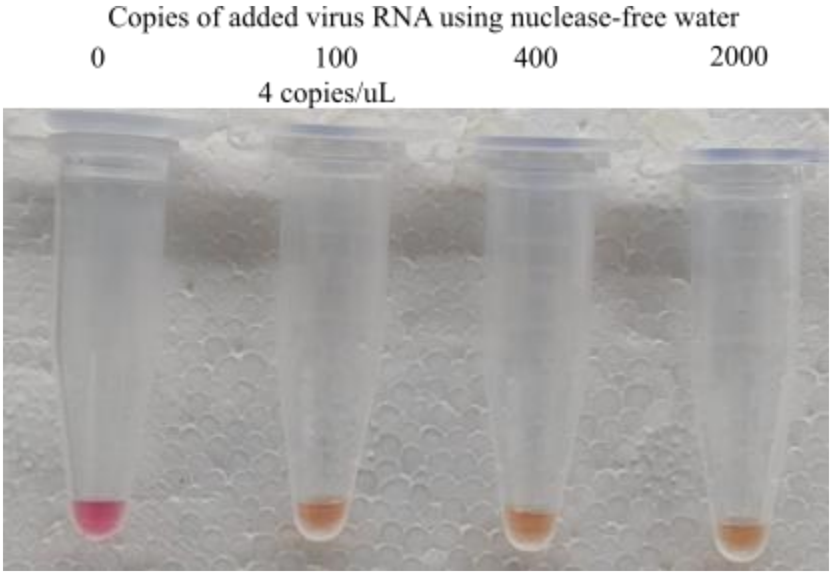

Fig S9. LAMP reaction results using commercial centrifuge (SciSpin – Micro, Cat. No:911315410035). After centrifugation, the LAMP protocol can effectively detect synthetic viral RNA down to 4 copies per µL in saliva.

6. A prototype kit with vials in several pouches.

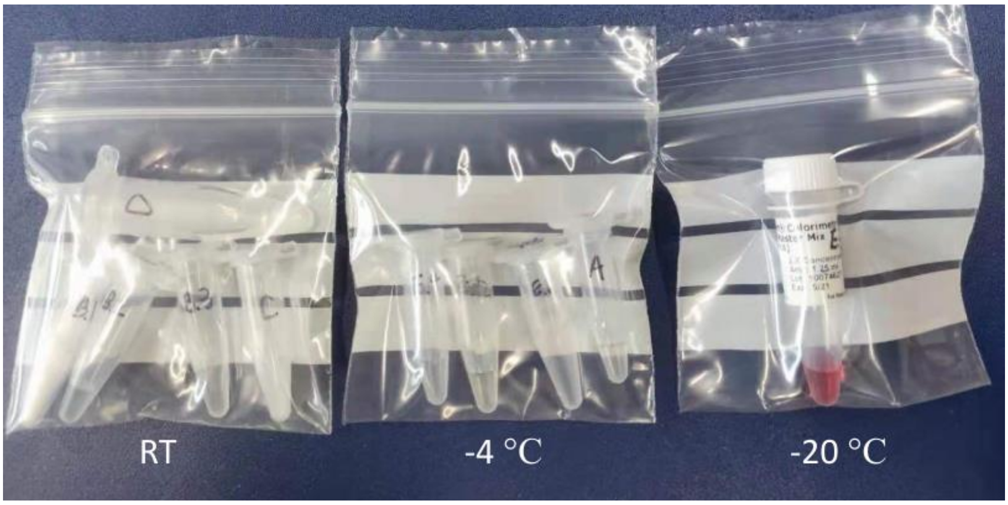

Fig S10. A prototype kit with vials in several pouches for room temperature, 4 °C and –20 °C storage.

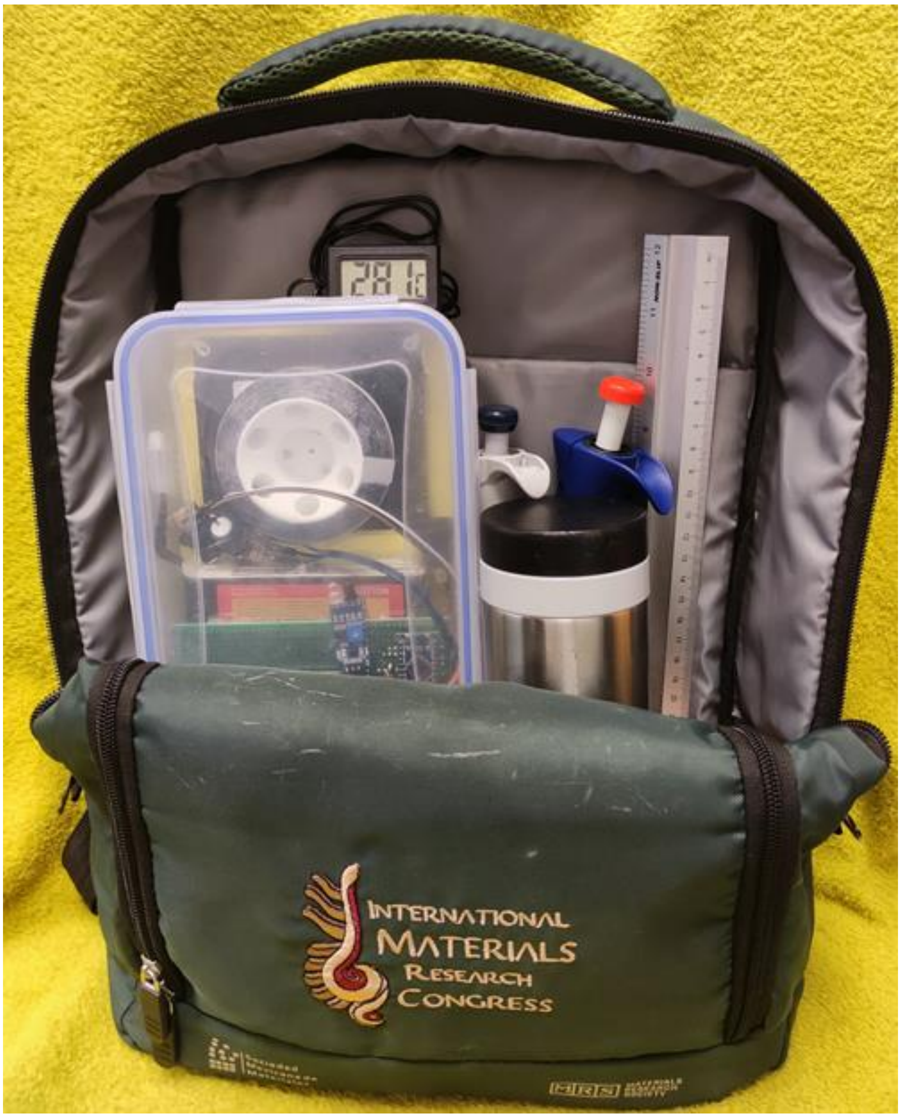

Fig S11. The $51 lab-in-a-backpack system pictured in a small backpack, fitting the CentriDrive centrifuge, a 1000 cm^3 cooler/freezer box for reagents under the centrifuge, a thermos, digital thermometer, two pipettes, and room for additional storage (ruler for scale).

Reference:

1. Manzoor M. Arduino with Infrared Sensor [cited 2021 Sep 18]. Available from: https://medium.com/illumination/arduino-with-infrared-sensor-48ad4415f320.
