# Supplementary figures and images for: End-to-end system for rapid and sensitive early-detection of SARS-CoV-2 for resource-poor and field-test environments using a $51 lab-in-a-backpack
Source: PLoS One. 2022 Jan 26;17(1):e0259886. doi: 10.1371/journal.pone.0259886 (PMC8791454; doi:10.1371/journal.pone.0259886)

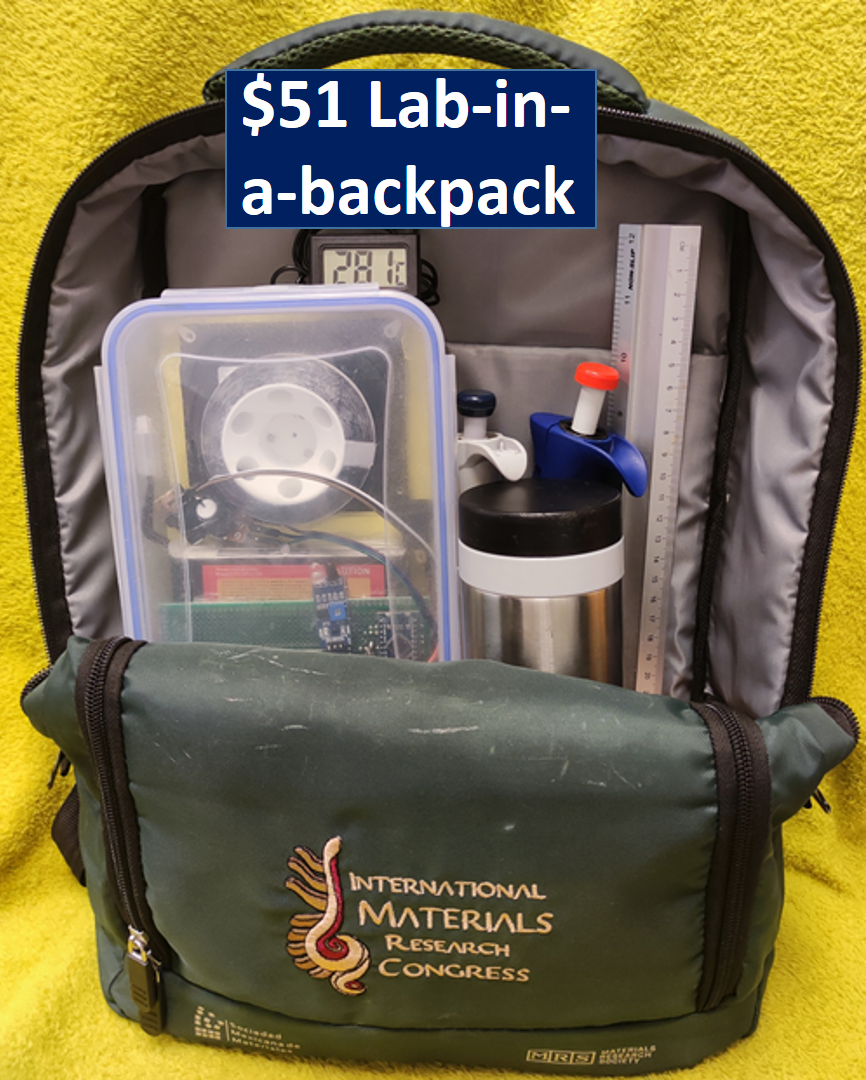

Supplement: S1 Graphical abstract — (TIF) [file pone.0259886.s002.tif]
